# Voxel-Based Morphometry in Women with Borderline Personality Disorder with and without Comorbid Posttraumatic Stress Disorder

**DOI:** 10.1371/journal.pone.0065824

**Published:** 2013-06-12

**Authors:** Inga Niedtfeld I, Lars Schulze, Annegret Krause-Utz, Traute Demirakca, Martin Bohus, Christian Schmahl

**Affiliations:** 1 Department of Psychosomatic Medicine, Central Institute of Mental Health Mannheim, Medical Faculty Mannheim/Heidelberg University, Mannheim, Germany; 2 Division of Clinical Psychology and Psychotherapy, Deptartment of Education and Psychology, Freie Universität Berlin, Berlin, Germany; 3 Department Neuroimaging, Central Institute of Mental Health Mannheim, Medical Faculty Mannheim/Heidelberg University, Mannheim, Germany; West China Hospital of Sichuan University, China

## Abstract

Patients with Borderline Personality Disorder (BPD) showed reduced volume of amygdala and hippocampus, but similar findings are evident in Posttraumatic Stress Disorder (PTSD). Applying voxel-based morphometry (VBM) in a larger cohort of patients with BPD, we sought to extend earlier findings of volume abnormalities in limbic regions and to evaluate the influence of co-occurring PTSD in BPD patients. We used voxel-based morphometry to study gray matter volume (GMV) in 60 healthy controls (HC) and 60 patients with BPD. Subgroup analyses on 53 patients concerning the role of co-occurring PTSD were conducted. Additionally, regression analyses were calculated to assess the relation between borderline symptom severity as well as dissociative experiences and GMV. Differences in local GMV between patients with BPD and HC were observed in the amygdale and hippocampus as well as in the fusiform and cingulate gyrus. Co-occurring PTSD was accompanied by increased GMV in the superior temporal gyrus and dorsolateral prefrontal cortex. Independent of co-occurring PTSD, severity of BPD symptoms predicted smaller GMV in the amygdala and dorsal ACC. Dissociation was positively related to GMV in the middle temporal gyrus. We could replicate earlier findings of diminished limbic GMV in patients with BPD and additionally show that patients with co-morbid PTSD feature increased GMV in prefrontal regions associated with cognitive control.

## Introduction

Borderline Personality Disorder (BPD) is a highly prevalent disorder [1]–[3] with instability in interpersonal relationships and self-image as well as emotion dysregulation as its core symptoms [4]. Earlier studies on brain structure in BPD found diminished gray matter volume (GMV) in amygdala, hippocampus, cingulate cortex, frontal lobe, and parietal cortex [5]–[17]. Based on this growing body of research, it was speculated that reduced volume of amygdala and hippocampus might be “biological markers” of BPD (for a meta-analysis, see [18]). However, reduced volume of the hippocampus and amygdala are also commonly observed in patients with Posttraumatic Stress Disorder (PTSD, for a meta-analysis, see [19]), and in women with a history of sexual abuse in childhood [20], [21]. Both conditions are also highly prevalent in patients with BPD. For instance, about fifty percent of BPD patients fulfill criteria for PTSD [22]. One may conclude that both disorders (BPD and PTSD) share common biological factors, and it is not clear whether these volume reductions are related to elevated stress levels or genetic factors [23], [24]. In sum, up until now, it remains unclear whether abnormalities in GMV of the limbic system are exclusively attributable to BPD.

A recent meta-analysis aimed to evaluate the influence of co-occurring PTSD in BPD patients and points to reduced bilateral hippocampal volumes in patients with BPD compared to healthy control subjects (HC). Importantly, these differences in GMV of the hippocampus were more pronounced for patients with BPD and co-morbid PTSD [25]. In both disorders, dissociation is a frequent psychopathological symptom [26], [27] and is more specifically defined as an altered state of consciousness causing impairments in body awareness, perception, and memory [4]. Dissociative symptoms are often triggered by either specific stimuli, emotional arousal or aversive tension [28], [29], providing a possible symptomatic link between both disorders.

Most studies on brain volume in BPD used manual tracing methods [5]–[7], [10]–[13], [15], thereby following an a priori region-of-interest approach that allows for precise detection of small volume differences. To our knowledge, there are only six studies available that report whole-brain results on GMV in BPD using voxel-based morphometry (VBM), which is a technique to conduct voxel-wise comparisons of and gray matter concentration (GMC) between groups of subjects, searching for structural differences within the whole brain [30]. The first study by Ruesch and colleagues [9] in twenty BPD patients found reduced GMV in the left amygdala compared to healthy control subjects (HC). The second study by Soloff et al. [14] compared 34 BPD patients to HC and observed changes in GMC in the hippocampus, amygdala, and ventral cingulate gyrus. Smaller GMV in the cingulate gyrus was associated with high depression, while smaller GMV in limbic and paralimbic regions was negatively correlated with impulsivity. In the third study, Völlm and colleagues [31] investigated GMV in seven male patients with BPD and six HC and found differences in orbitofrontal cortex, middle frontal gyrus, precentral and postcentral gyrus, temporal pole, and inferior and superior parietal cortex, which were negatively correlated to trait impulsivity. In the fourth study, Brunner and colleagues [16] compared 20 adolescent patients with BPD to patients with other psychiatric disorders and HC. BPD patients showed reduced GMV in the dorsolateral prefrontal cortex (DLPFC) and orbitofrontal cortex compared to HC, but there were no significant GMV differences compared to the clinical control group, which also showed smaller DLPFC volumes than HC. In the fifth study by Soloff and colleagues [17], 68 patients (male and female) with BPD were investigated. The authors found reduced GMC in the insula, orbitofrontal gyrus and middle superior temporal cortex, which were associated with suicidal behavior in BPD. In the most recent study, Kuhlmann and colleagues [32] found reduced gray matter in 30 female patients with BPD in the hippocampus, and increased volume in the hypothalamus compared to 30 healthy participants, but no significant alterations were found in the amygdala or anterior cingulate cortex (ACC). Hypothalamic volume correlated positively with the self-ratings of past traumatization in patients with BPD. However, no differences were found between subgroups of BPD patients with comorbid PTSD (n = 9) and BPD patients without PTSD (n = 21). Regression analyses did not reveal any significant correlations with clinical variables (e.g. BPD symptom severity as assessed by the Borderline Symptom List). In total, available evidence regarding GMV differences in the limbic system in BPD is rather inconclusive at the present time. Moreover, most of the previous studies included rather small sample sizes, which did not allow for controlling the role of co-occurring PTSD in individuals with BPD.

In the present study, we sought to extend earlier findings of volume alterations in limbic regions by applying voxel-based morphometry (VBM) in a larger cohort of patients with BPD. Moreover, we were interested in GMV differences in patients with BPD and co-occurring PTSD. Additionally, we investigated whether changes in GMV are related to borderline symptom severity as well as dissociative experiences.

## Methods

Structural imaging data of 60 women with BPD according to DSM-IV [4] without psychotropic medication (age *M* = 29.67, *SD* = 8.06) and 60 healthy women (age *M* = 28.5, *SD* = 7.49; *T*_(118)_ = 0.763, p = .447) were collected on a 3T MRI scanner (TRIO, Siemens Medical Systems, Erlangen, Germany) between 2007 and 2011 at the Central Institute of Mental Health in Mannheim. Data can be obtained per request. The T1-weighted high-resolution structural scan was acquired using 3-D magnetization-prepared-rapid-acquisition-gradient-echo (176 sagittal slices, voxel size 1×1×1 mm, 256 mm field of view, repetition time 1570 ms, echo time 2.75 ms, flip angle 15°, echo spacing = 8.2 ms, inversion time 800 ms). To assess psychiatric disorders, all participants were rated by trained psychologists with semi-structured interviews (Structured Clinical Interview for Axis I disorders, SCID-I, [33]; International Personality Disorder Examination, IPDE [34]). Inter-rater reliability for IPDE/SCID was assessed using exemplary video interviews by master trainers, which were then rated by all individual raters involved in the study. This resulted in a sufficient inter-rater reliability (κ = .77). Exclusion criteria for all participants were severe medical or neurological illnesses, organic brain disease, mental retardation, medical history of skull- and/or brain-damage, pregnancy, left-handedness, pieces of metal in the body, claustrophobia, psychotropic medication two weeks prior to the study, as well as substance abuse or dependency during the last year prior to the study. Reliable data on PTSD co-occurrence was available for 53 BPD patients. 21 BPD patients met PTSD diagnosis (“BPD+PTSD”) and 32 did not fulfill the diagnosis (“BPD-PTSD”). Traumatic events comprised physical or sexual abuse.

A total of 42 BPD patients completed the Borderline Symptom List (BSL) [35] measuring BPD symptom severity, and the German adaptation of the Dissociative Experience Scale (Fragebogen zu Dissoziativen Symptomen, FDS) [36]. Patients with BPD+PTSD had higher FDS scores (M = 26.7, SD = 15.8) than patients in the BPD-PTSD group (M = 19.5, SD = 8.8) on a descriptive level, which did not reach statistical significance (T(24) = 1.493, p = .149). Severity of borderline symptoms was comparable between the BPD+PTSD (M = 189.6, SD = 39.4) and the BPD-PTSD group (M = 181.1, SD = 55.3; T(28) = 0.469, p = .642). All participants gave written informed consent to a study protocol according to the Declaration of Helsinki and approved by the ethics committee of the University of Heidelberg. We only included participants with full capacity to consent. Capacity to consent was established during a clinical interview.

For MRI analysis, we applied standard procedures implemented in the VBM8 toolbox (http://dbm.neuro.uni-jena.de/vbm/), implemented as a toolbox in SPM8 (Wellcome Department of Cognitive Neurology, London; www.fil.ion.ucl.ac.uk/spm). Data preprocessing consisted of segmentation into gray and white matter images, as well as a bias correction for magnetic field inhomogeneities. Additionally, Hidden Markov Random Fields were applied to increase the signal-to-noise ratio [35]. All resulting native GM and WM images were registered to a template provided by the International Consortium of Brain Mapping and a diffeomorphic image registration algorithm (DARTEL) [36] was used for spatially normalizing GM images into MNI space. DARTEL is a nonlinear algorithm to transform native images in stereotactic space which has proven to be suitable for morphometry studies [37]. Finally, the modulated normalized gray matter maps (m0wrp1*), depicting the absolute amount regional GMV corrected for individual brain sizes, were smoothed with a standard 10 mm full-width-at-half-maximum (FWHM) isotropic Gaussian kernel and used for further statistical analyses of differences in GMV between both groups.

Group differences in local GMV between HC and BPD were analyzed using two-sample t-tests. We also investigated subgroups of BPD patients without (BPD-PTSD, n = 31) and with current PTSD (BPD+PTSD, n = 21). Additionally, we used regression analyses within the subgroup of 42 BPD patients for whom questionnaires were available entering trait dissociation scores (FDS) [38] as well as dimensional scores of borderline symptom severity (BSL) [39] as regressors. For significant clusters, correlation coefficients were calculated between GMV of the peak-voxel and questionnaire scores using custom statistical software (SPSS, Rel. 15.0.1. 2006. Chicago: SPSS Inc.). Furthermore, stepwise regression analyses were conducted to investigate whether comorbid PTSD explains additional variance in this context. We decided not to include healthy controls in the regression analyses due to floor effects. In all analyses, we used an absolute threshold of 0.2 (probability for the presence of gray matter) to prevent effects located at the border regions of the tissue maps. Furthermore, in all analyses age was used as a covariate of no interest.

Region of Interest (ROI) Analyses were conducted for the bilateral amygdala, hippocampus and ACC. Therefore, anatomical masks defined by the Automated Anatomical Labeling software were used [40]. Since we were also interested in differences in prefrontal regions, we used the peak voxel in DLPFC which was found by Brunner and colleagues [16] with a 10 mm sphere at the MNI-coordinate [−18, 63, 24].

To reduce the possibility of type-I errors, we chose the following procedures: For a-priori-defined ROI analyses we chose a statistical threshold of p(FWE)<.05. For whole-brain analyses, we used the cluster extent correction procedure implemented in spm8, which computes the number of expected voxels per cluster according to random field theory [41]. More specifically, we combined a cluster-defining height threshold (set at p<.001 uncorrected in the present study) with an empirically determined extent threshold (expected number of voxels per cluster) [42]; [43]. Importantly, cluster sizes are known to vary with local roughness of the provided images. Thus, non-stationary random field theory procedures were used for cluster-size statistics adjusting cluster sizes depending on the local smoothness of the data [41]. For the two-sample t-test (BPD vs. HC), the minimum cluster size was determined to be 189 adjacent voxels, for the subgroup analysis (two-sample t-test BPD+PTSD vs. BPD-PTSD) 179 voxels, and for regression analyses (BSL and FDS) 158 and 146 voxels, respectively.

## Results

**Figure 1:**
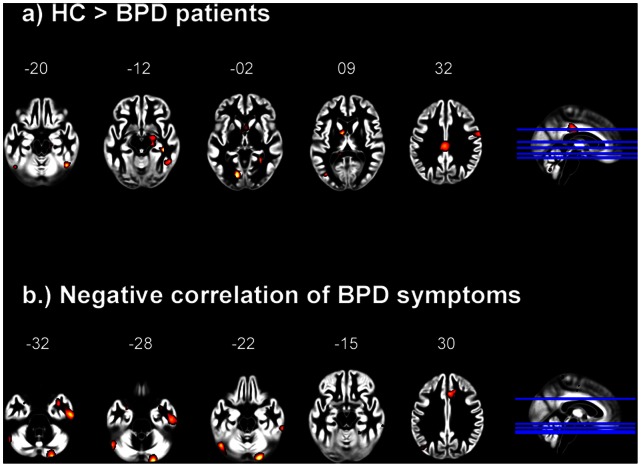
Whole-brain maps illustrate smaller gray matter volumes in patients with borderline personality disorders compared to healthy controls (1a) and negative correlations between gray matter volume and the severity of BPD symptoms (1b). For visualization purposes, the statistical maps were thresholded at T>2.5. Size and location of clusters are reported in Table 1 and 2.

Voxel-based analyses revealed significant differences in local GMV between patients with BPD and HC. In the ROIs, patients with BPD showed smaller volumes in the right amygdala, right hippocampus and BA 23/cingulate gyrus (see Table 1 and Figure 1a). The whole-brain analysis illustrated less GMV compared to HC in the fusiform gyrus (BA37)/inferior temporal gyrus and lingual gyrus (see Figure 1a and Table 2 for more details). The reverse contrast (BPD>HC) revealed no significant results.

**Table 1:** Results of ROI analyses of gray matter volume.

Test and Contrast	Brodmann Area	AAL	k	p(FWE)	p(unc)	equivZ	MNI [x y z]		
ROI Analyses: HC>BPD									
	Amygdala	Amygdala (right)	494	0.016	0.001	3.07	23	−9	−12
		Hippocampus (left)	2009	0.074	0.002	2.944	−12	−39	9
	BA 35	Hippocampus (right)	2148	0.034	0.001	3.212	20	−23	−14
	BA 23	Cingulate gyrus	9100	0.052	0.000	3.41	−2	−26	32
ROI Analyses: BPD+PTSD>BPD-PTSD									
	BA 10	dlPFC	388	0.039	0.002	2.878	−12	63	18
ROI Analyses: Regression Analysis BSL (negative correlation)									
		Amygdala (left)	579	0.053	0.004	2.672	−26	5	−29
	Amygdala	Amygdala (right)	494	0.096	0.009	2.357	18	−6	−15
	BA 32	dorsal ACC	3253	0.04	0.000	3.303	6	23	30

ACC = anterior cingulate cortex; DLPFC = dorsolateral prefrontal cortex; k = number of voxels within ROI masks.

**Table 2:** Whole Brain Results of voxel-based analyses of gray matter volume.

Test and Contrast	Brodmann Area	AAL	k	p(FWE)	p(unc)	equivZ	MNI [x y z]		
Two-Sample T-Test: HC>BPD									
		Lingual gyrus	375	0.296	0.000	3.85	−14	−78	−2
	BA 37	Fusiform & inferior temporal gyrus	299	0.612	0.000	3.54	47	−58	−20
Two-Sample T-Test: BPD+PTSD>BPD-PTSD									
	BA 22	Superior temporal gyrus	241	0.320	0.000	3.85	−65	−34	12
Regression Analysis BSL (negative correlation)									
	BA 18	Cerebellum	377	0.382	0.000	3.83	21	−88	−26
	BA 37	Cerebellum	405	0.686	0.000	3.54	−48	−63	−24
	BA 19	Fusiform gyrus		0.806	0.000	3.42	−44	−69	−20
		Inferior temporal gyrus	237	0.712	0.000	3.52	57	−15	−32
Regression Analysis FDS (positive correlation)									
		Middle temporal gyrus	159	0.792	0.000	3.46	50	−49	10

ACC = anterior cingulate cortex; k = cluster size.

**Figure 2:**
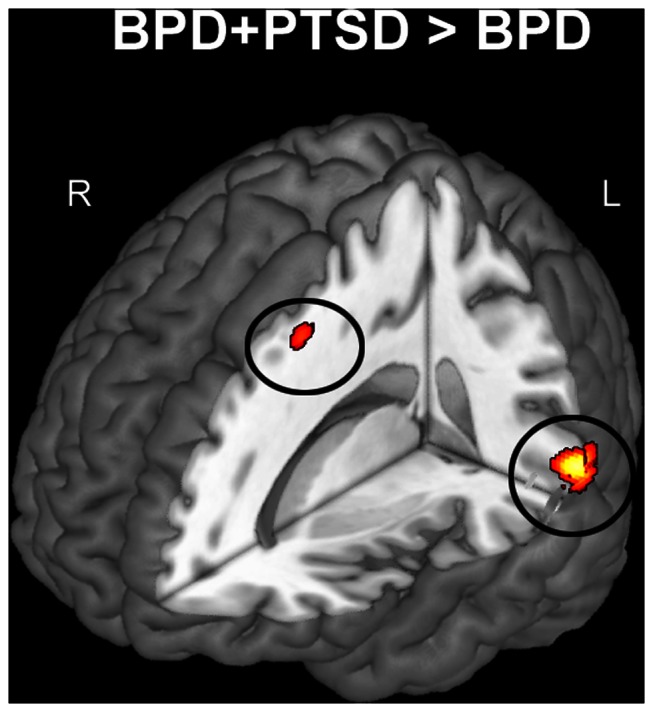
Whole-brain maps illustrate smaller gray matter volumes in patients with borderline personality disorder (BPD-PTSD) compared to patients with borderline personality disorders and co-occurring PTSD (BPD+PTSD). For visualization purposes, the statistical maps were thresholded at T>2.5. Size and location of clusters are reported in Table 1 and 2.

Comparing BPD-PTSD to BPD+PTSD, patients with co-occurring PTSD showed more GMV in superior temporal gyrus (BA22) and DLPFC (see Figure 2). No significant group differences in hippocampal or amygdala GMV were found between BPD patients with vs. without co-morbid PTSD.

Regression analyses with trait dissociation scores (FDS) showed higher GMV in middle temporal gyrus in patients with high scores of trait dissociation (r = .34). Patients with high borderline symptom severity showed less GMV in the cerebellum (visual association area), as well as in the fusiform gyrus/inferior temporal gyrus (see Table 2 and Figure 1b). A significant negative correlation with borderline symptom severity was observed within the ROI-analyses of the dorsal ACC (r = –.56), and a statistical trend for the bilateral amygdala (r = –.49) (see Table 1 and Figure 1b). Furthermore, stepwise multiple regression analyses confirmed borderline symptom severity as the strongest predictor for GMV in the left amygdala (beta = −0.36, p<0.05), whereas co-morbid PTSD did not explain incremental variance (beta = −0.08, p = .66).

## Discussion

In this study, we used VBM to extend findings on gray matter volume (GMV) in patients with BPD. Moreover, we investigated the influence of co-occurring PTSD, as well as the role of borderline symptom severity and dissociative experiences. We were able to replicate reduced GMV in right amygdala, right hippocampus, and cingulate cortex in BPD [7], [14], [18], [32]. Additionally, we found diminished GMV in the fusiform and inferior temporal gyrus in BPD patients, which was also observed in three other studies using VBM in BPD [9], [14], [32]. Deviations in amygdala, hippocampus and fusiform gyrus were observed not only in volumetric studies, but also in fMRI studies in BPD [44]–[46], presumably reflecting affective instability or problems with emotion regulation. While our finding of posterior cingulate gyrus volume reduction in BA 23 corresponds to the study by Hazlett and colleagues [12], whereas other studies in BPD have found reduced volumes in more caudal portions of the ACC, such as BA 24 and BA 32 [14].

Interestingly, a recent study in healthy adults provides a possible explanation for the relation of structural and functional abnormalities in limbic regions in BPD patients [21]. The authors demonstrated structural and functional alterations in healthy subjects with childhood maltreatment, which are strikingly similar to some findings in BPD research. First, the authors found reduced GMV in the hippocampus, orbitofrontal cortex, and ACC dependent on the severity of adverse events in childhood. Second, childhood maltreatment in healthy adults was also associated with higher amygdala responsiveness when viewing threat-related facial expressions [21]. This leads to the assumption that alterations in limbic brain regions could be mediators between adverse events in childhood and the development of psychiatric disorders like BPD, PTSD or major depression [47]. Nonetheless, it was argued that the co-occurrence of early childhood traumatization along with reduced abilities to regulate emotions and heightened impulsivity might be more specific for the development of BPD [48].

Investigating the influence of PTSD, we compared patients with BPD to patients with both BPD and PTSD. Importantly, the subgroups (21 vs. 32 patients) were large enough to ensure sufficient statistical power. The subgroup of patients with BPD and PTSD showed increased volumes in DLPFC (BA9), and superior temporal gyrus (BA22) compared to BPD patients without PTSD. To date, there is only one study showing reduced DLPFC (BA9) volumes in adolescents with BPD [16], but there are a number of studies pointing to reduced prefrontal efficiency [45], [49]–[51]. Since DLPFC activity was found to be implicated in the suppression of unwanted memories [52], one could tentatively claim that volume differences are driven by use-dependent brain plasticity [53]. This would imply that co-morbid PTSD is characterized by frequent inhibition of emotions via the DLPFC [27], resulting in increased GMV. However, since our results are correlational, an other possible explanation is that individuals with a large DLPFC could be more vulnerable to develop PTSD. We found no significant differences in GMV between the subgroups in the amygdala or hippocampus.

Further investigating the influence of symptom levels, we found that high trait dissociation scores coincide with increased GMV in middle temporal gyrus. This finding corresponds to the temporal lobe hypothesis of dissociation. Studies in PTSD, Depersonalization Disorder as well as literature on temporal lobe epilepsy point to a connection between brain function in the middle temporal gyri and dissociative symptoms [54], [55]. One could reason that frequent states of dissociation may be connected to volume changes in temporal gyri.

Finally, we found that high severity of borderline symptoms was correlated to reduced GMV in the visual association cortex, the fusiform gyrus and inferior temporal gyrus, which are implicated in face perception. Correspondingly, most functional imaging studies on emotion processing in BPD found increased activity in these brain areas [44], [45], [56]. One possible explanation was first mentioned by Herpertz and colleagues [44] concerning the modulation of perceptual areas by back-projections from the amygdala, resulting in increased sensitivity to emotional stimuli. Accordingly, borderline severity was correlated to GMV reduction in the left amygdala, which was statistically independent from co-morbid PTSD.

However, since we did not investigate patients with PTSD alone, our conclusions are restricted to patients with BPD and co-morbid PTSD. Furthermore, we investigated only patients with BPD and current PTSD, and can not exclude the possibility that some patients had previous lifetime-diagnoses of PTSD. Therefore, it is important to note that one should not draw conclusions from the absence of significant differences between BPD+PTSD and BPD-PTSD. Future studies should include a group of patients with PTSD to examine the specificity of our results. Unfortunately, we also have no differentiated information whether participants in in the non-PTSD group also experienced traumatic events. Although it is safe to assume that most of the BPD patients did experience adverse and traumatic events in childhood (for a Review, see [57]), it remains unclear whether group differences are indeed primarily attributable to PTSD. Another limitation of this study is the lack of rigorous statistical correction for multiple comparisons (i.e., FWE) and a relatively small smoothing kernel. However, four out of the six other studies using VBM in BPD chose similar or even more liberal correction procedures [9], [17], [31], [32]. Nevertheless, those correction procedures would have reduced the possibility of a type I error.

In this study we could replicate results from previous studies on GMV loss in limbic regions (amygdala, hippocampus) in BPD. Patients with co-occurring PTSD as well as patients with high dissociation showed increased brain volume in superior and middle temporal gyri. Independent of co-occurring PTSD, the GMV of the left amygdala was shown to be strongly correlated with the severity of BPD symptoms.

## References

[1] Skodol AE, Gunderson JG, Pfohl B, Widiger TA, Livesley WJ, et al (2002) The borderline diagnosis I: Psychopathology comorbidity, and personaltity structure. Biological psychiatry 51: 936–950. 12062877 10.1016/s0006-3223(02)01324-0

[2] Grant BF, Chou SP, Goldstein RB, Huang B, Stinson FS, et al (2008) Prevalence, correlates, disability, and comorbidity of DSM-IV borderline personality disorder: results from the Wave 2 National Epidemiologic Survey on Alcohol and Related Conditions. Journal of Clinical Psychiatry 69: 533–545. 18426259 10.4088/jcp.v69n0404 PMC2676679

[3] Korzekwa MI, Dell PF, Links PS, Thabane L, Webb SP (2008) Estimating the prevalence of borderline personality disorder in psychiatric outpatients using a two-phase procedure. Comprehensive Psychiatry 49: 380–386. 18555059 10.1016/j.comppsych.2008.01.007

[4] Association AP (1994) Diagnostic and statistical manual of mental disorders (DSM-IV); Association AP, editor. Washington, DC, USA: American Psychiatric Association. - p.

[5] Lyoo IK, Han MH, Cho DY (1998) A brain MRI study in subjects with borderline personality disorder. Journal of Affective Disorders 50: 235–243. 9858082 10.1016/s0165-0327(98)00104-9

[6] Driessen M, Herrmann J, Stahl K, Zwaan M, Meier S, et al (2000) Magnetic resonance imaging volumes of the hippocampus and the amygdala in women with borderline personality disorder and early traumatization. Archives of General Psychiatry 57: 1115–1122. 11115325 10.1001/archpsyc.57.12.1115

[7] Tebartz van Elst L, Hesslinger B, Thiel T, Geiger E, Haegele K, et al (2003) Frontolimbic brain abnormalities in patients with borderline personality disorder: a volumetric magnetic resonance imaging study. Biological psychiatry 54: 163–171. 12873806 10.1016/s0006-3223(02)01743-2

[8] Schmahl CG, Vermetten E, Elzinga BM, Douglas Bremner J (2003) Magnetic resonance imaging of hippocampal and amygdala volume in women with childhood abuse and borderline personality disorder. Psychiatry research 122: 193–198. 12694893 10.1016/s0925-4927(03)00023-4

[9] Rüsch N, van Elst LT, Ludaescher P, Wilke M, Huppertz HJ, et al (2003) A voxel-based morphometric MRI study in female patients with borderline personality disorder. NeuroImage 20: 385–392. 14527598 10.1016/s1053-8119(03)00297-0

[10] Brambilla P, Soloff PH, Sala M, Nicoletti MA, Keshavan MS, et al (2004) Anatomical MRI study of borderline personality disorder patients. Psychiatry research 131: 125–133. 15313519 10.1016/j.pscychresns.2004.04.003

[11] Irle E, Lange C, Sachsse U (2005) Reduced size and abnormal asymmetry of parietal cortex in women with borderline personality disorder. Biological psychiatry 57: 173–182. 15652877 10.1016/j.biopsych.2004.10.004

[12] Hazlett EA, New AS, Newmark R, Haznedar MM, Lo JN, et al (2005) Reduced anterior and posterior cingulate gray matter in borderline personality disorder. Biological Psychiatry 58: 614–623. 15993861 10.1016/j.biopsych.2005.04.029

[13] Zetzsche T, Frodl T, Preuss UW, Schmitt G, Seifert D, et al (2006) Amygdala volume and depressive symptoms in patients with borderline personality disorder. Biological Psychiatry 60: 302–310. 16476409 10.1016/j.biopsych.2005.11.020

[14] Soloff P, Nutche J, Goradia D, Diwadkar V (2008) Structural brain abnormalities in borderline personality disorder: a voxel-based morphometry study. Psychiatry research 164: 223–236. 19019636 10.1016/j.pscychresns.2008.02.003 PMC3286221

[15] Schmahl C, Berne K, Krause A, Kleindienst N, Valerius G, et al (2009) Hippocampus and amygdala volumes in patients with borderline personality disorder with or without posttraumatic stress disorder. Journal of psychiatry & neuroscience: JPN 34: 289–295. 19568480 PMC2702446

[16] Brunner R, Henze R, Parzer P, Kramer J, Feigl N, et al (2010) Reduced prefrontal and orbitofrontal gray matter in female adolescents with borderline personality disorder: is it disorder specific? NeuroImage 49: 114–120. 19660555 10.1016/j.neuroimage.2009.07.070

[17] Soloff PH, Pruitt P, Sharma M, Radwan J, White R, et al (2012) Structural brain abnormalities and suicidal behavior in borderline personality disorder. Journal of Psychiatric Research 46: 516–525. 22336640 10.1016/j.jpsychires.2012.01.003 PMC3307855

[18] Nunes PM, Wenzel A, Borges KT, Porto CR, Caminha RM, et al (2009) Volumes of the hippocampus and amygdala in patients with borderline personality disorder: a meta-analysis. Journal of Persality Disorders 23: 333–345. 10.1521/pedi.2009.23.4.333 19663654

[19] Karl A, Schaefer M, Malta LS, Dorfel D, Rohleder N, et al (2006) A meta-analysis of structural brain abnormalities in PTSD. Neuroscience and biobehavioral reviews 30: 1004–1031. 16730374 10.1016/j.neubiorev.2006.03.004

[20] Stein MB, Koverola C, Hanna C, Torchia MG, McClarty B (1997) Hippocampal volume in women victimized by childhood sexual abuse. Psychological Medicine 27: 951–959. 9234472 10.1017/s0033291797005242

[21] Dannlowski U, Stuhrmann A, Beutelmann V, Zwanzger P, Lenzen T, et al (2012) Limbic scars: long-term consequences of childhood maltreatment revealed by functional and structural magnetic resonance imaging. Biological Psychiatry 71: 286–293. 22112927 10.1016/j.biopsych.2011.10.021

[22] Lieb K, Zanarini MC, Schmahl C, Linehan MM, Bohus M (2004) Borderline personality disorder. Lancet 364: 453–461. 15288745 10.1016/S0140-6736(04)16770-6

[23] Schmahl C, Berne J, Krause A, Kleindienst N, Valerius G, et al (2009) Hippocampal and amygdala volumes in borderline personality disorder with and without posttraumatic stress disorder. Journal of Psychiatry and Neuroscience 34: 289–295. 19568480 PMC2702446

[24] Krause-Utz A, Schmahl C (2010) Neurobiological Differentiation Between Borderline Patients With and Without Post-traumatic Stress Disorder. European Psychiatric Review 3: 63–68.

[25] Rodrigues E, Wenzel A, Ribeiro MP, Quarantini LC, Miranda-Scippa A, et al (2011) Hippocampal volume in borderline personality disorder with and without comorbid posttraumatic stress disorder: a meta-analysis. Eur Psychiatry 26: 452–456. 20933369 10.1016/j.eurpsy.2010.07.005

[26] Zanarini MC, Ruser TF, Frankenburg FR, Hennen J, Gunderson JG (2000) Risk factors associated with the dissociative experiences of borderline patients. Journal of Nervous and Mental Disease 188: 26–30. 10665457 10.1097/00005053-200001000-00005

[27] Lanius RA, Vermetten E, Loewenstein RJ, Brand B, Schmahl C, et al (2010) Emotion modulation in PTSD: Clinical and neurobiological evidence for a dissociative subtype. American Journal of Psychiatry 167: 640–647. 20360318 10.1176/appi.ajp.2009.09081168 PMC3226703

[28] Stiglmayr C, Gratwohl T, Bohus M (2001) States of Aversive Tension in Patients with Borderline Personality Disorder: A Controlled Field Study. In: Fahrenberg J, Myrtek M, editors. Seattle: Hogrefe & Huber. 135–141.

[29] Stiglmayr CE, Ebner-Priemer UW, Bretz J, Behm R, Mohse M, et al (2008) Dissociative symptoms are positively related to stress in borderline personality disorder. Acta psychiatrica Scandinavica 117: 139–147. 18028248 10.1111/j.1600-0447.2007.01126.x

[30] Ashburner J, Friston KJ (2000) Voxel-Based Morphometry–The Methods. Neuroimage 11: 805–821. 10860804 10.1006/nimg.2000.0582

[31] Vollm BA, Zhao L, Richardson P, Clark L, Deakin JF, et al (2009) A voxel-based morphometric MRI study in men with borderline personality disorder: preliminary findings. Crim Behav Ment Health 19: 64–72. 19172640 10.1002/cbm.716

[32] Kuhlmann A, Bertsch K, Schmidinger I, Thomann PA, Herpertz SC (2013) Morphometric differences in central stress-regulating structures between women with and without borderline personality disorder. J Psychiatry Neurosci 38: 129–137. 22909445 10.1503/jpn.120039 PMC3581593

[33] Wittchen HU, Wunderlich U, Gruschwitz S (1997) SKID. Strukturiertes Klinisches Interview für DSM-IV Achse I. Göttingen: Hogrefe. - p.

[34] Loranger AW (1999) International Personality Disorder Examination (IPDE): DSM-IV and ICD-10 modules. Odessa: Psychological Assessment Resources. - p.

[35] Zhang Y, Brady M, Smith S (2001) Segmentation of brain MR images through a hidden Markov random field model and the expectation-maximization algorithm. IEEE Transactions on Medical Imaging 20: 45–57. 11293691 10.1109/42.906424

[36] Ashburner J (2007) A fast diffeomorphic image registration algorithm. Neuroimage 38: 95–113. 17761438 10.1016/j.neuroimage.2007.07.007

[37] Klein A, Andersson J, Ardekani BA, Ashburner J, Avants B, et al (2009) Evaluation of 14 nonlinear deformation algorithms applied to human brain MRI registration. Neuroimage 46: 786–802. 19195496 10.1016/j.neuroimage.2008.12.037 PMC2747506

[38] Freyberger HJ, Spitzer C, Stieglitz RD (1999) Fragebogen zu dissoziativen Symptomen (FDS). Ein Selbstbeurteilungsverfahren zur syndromalen Diagnostik dissoziativer Phaenomene. Deutsche Adaptation der Dissociative Experience Scale (DES) von E.Bernstein und F.W.Putnam. Bern: Verlag Hans Huber. - p.

[39] Bohus M, Limberger MF, Frank U, Chapman AL, Kuhler T, et al (2007) Psychometric properties of the Borderline Symptom List (BSL). Psychopathology 40: 126–132. 17215599 10.1159/000098493

[40] Tzourio-Mazoyer N, Landeau B, Papathanassiou D, Crivello F, Etard O, et al (2002) Automated anatomical labeling of activations in SPM using a macroscopic anatomical parcellation of the MNI MRI single-subject brain. Neuroimage 15: 273–289. 11771995 10.1006/nimg.2001.0978

[41] Hayasaka S, Nichols TE (2004) Combining voxel intensity and cluster extent with permutation test framework. Neuroimage 23: 54–63. 15325352 10.1016/j.neuroimage.2004.04.035

[42] Nenadic I, Smesny S, Schlosser RG, Sauer H, Gaser C (2010) Auditory hallucinations and brain structure in schizophrenia: voxel-based morphometric study. Br J Psychiatry 196: 412–413. 20435970 10.1192/bjp.bp.109.070441

[43] Kluetsch RC, Schmahl C, Niedtfeld I, Densmore M, Calhoun VD, et al.. (2012) Alterations in Default Mode Network Connectivity During Pain Processing in Borderline Personality DisorderDefault Mode Network, Pain Processing, and BPD. Archives of General Psychiatry: 1–11. 10.1001/archgenpsychiatry.2012.476 PMC4429518 22637967

[44] Herpertz SC, Dietrich TM, Wenning B, Krings T, Erberich SG, et al (2001) Evidence of abnormal amygdala functioning in borderline personality disorder: a functional MRI study. Biological psychiatry 50: 292–298. 11522264 10.1016/s0006-3223(01)01075-7

[45] Koenigsberg HW, Fan J, Ochsner KN, Liu X, Guise KG, et al (2009) Neural correlates of the use of psychological distancing to regulate responses to negative social cues: a study of patients with borderline personality disorder. Biological psychiatry 66: 854–863. 19651401 10.1016/j.biopsych.2009.06.010 PMC2821188

[46] Niedtfeld I, Schulze L, Kirsch P, Herpertz SC, Bohus M, et al (2010) Affect regulation and pain in borderline personality disorder: a possible link to the understanding of self-injury. Biological psychiatry 68: 383–391. 20537612 10.1016/j.biopsych.2010.04.015

[47] Gilbert R, Widom CS, Browne K, Fergusson D, Webb E, et al (2009) Burden and consequences of child maltreatment in high-income countries. Lancet 373: 68–81. 19056114 10.1016/S0140-6736(08)61706-7

[48] Crowell SE, Beauchaine TP, Linehan MM (2009) A biosocial developmental model of borderline personality: Elaborating and extending Linehan's theory. Psychological bulletin 135: 495–510. 19379027 10.1037/a0015616 PMC2696274

[49] Sala M, Caverzasi E, Lazzaretti M, Morandotti N, De Vidovich G, et al (2011) Dorsolateral prefrontal cortex and hippocampus sustain impulsivity and aggressiveness in borderline personality disorder. Journal of Affective Disorders 131: 417–421. 21211852 10.1016/j.jad.2010.11.036

[50] O'Neill A, Frodl T (2012) Brain structure and function in borderline personality disorder. Brain Struct Funct 217: 767–782. 22252376 10.1007/s00429-012-0379-4

[51] New AS, Hazlett EA, Buchsbaum MS, Goodman M, Mitelman SA, et al (2007) Amygdala-prefrontal disconnection in borderline personality disorder. Neuropsychopharmacology 32: 1629–1640. 17203018 10.1038/sj.npp.1301283

[52] Anderson MC, Ochsner KN, Kuhl B, Cooper J, Robertson E, et al (2004) Neural systems underlying the suppression of unwanted memories. Science 303: 232–235. 14716015 10.1126/science.1089504

[53] Trachtenberg JT, Chen BE, Knott GW, Feng G, Sanes JR, et al (2002) Long-term in vivo imaging of experience-dependent synaptic plasticity in adult cortex. Nature 420: 788–794. 12490942 10.1038/nature01273

[54] Lanius RA, Williamson PC, Boksman K, Densmore M, Gupta M, et al (2002) Brain activation during script-driven imagery induced dissociative responses in PTSD: a functional magnetic resonance imaging investigation. Biological psychiatry 52: 305–311. 12208637 10.1016/s0006-3223(02)01367-7

[55] Simeon D, Guralnik O, Hazlett EA, Spiegel-Cohen J, Hollander E, et al (2000) Feeling unreal: a PET study of depersonalization disorder. American Journal of Psychiatry 157: 1782–1788. 11058475 10.1176/appi.ajp.157.11.1782

[56] Guitart-Masip M, Pascual JC, Carmona S, Hoekzema E, Berge D, et al (2009) Neural correlates of impaired emotional discrimination in borderline personality disorder: an fMRI study. Progress in neuro-psychopharmacology & biological psychiatry 33: 1537–1545. 19748540 10.1016/j.pnpbp.2009.08.022

[57] Ball JS, Links PS (2009) Borderline personality disorder and childhood trauma: evidence for a causal relationship. Current psychiatry reports 11: 63–68. 19187711 10.1007/s11920-009-0010-4

